# How have casemix, cost and hospital stay of inpatients in the last year of life changed over the past decade? Evidence from Italy

**DOI:** 10.1016/j.healthpol.2021.06.005

**Published:** 2021-08

**Authors:** Paolo Berta, Pietro Giorgio Lovaglio, Stefano Verzillo

**Affiliations:** a CRISP - Interuniversity Research Centre on Public Services, University of Milano Bicocca, Milan, Italy; b Department of Statistics and Quantitative Methods, University of Milano Bicocca, Milan, Italy; c European Commission, Joint Research Centre (JRC), Ispra Italy

**Keywords:** End-of-life, Healthcare services, Healthcare expenditure, Chronic care, Quantile regression

## Abstract

Healthcare utilisation and expenditure are highly concentrated in hospital inpatient services, in particular in end-of-life care with the peak occurring in the very last year of life, regardless of patient age. Few scientific studies have investigated hospital costs and stays of patients at the end of life, and even fewer studies have analysed their evolution over time. In this paper, we exploit hospitalisation data for the Lombardy region of Italy with the aim of studying the evolution of hospital casemix, costs and stays of chronic patients, and compare the last year of life of two cohorts of patients who died in 2005 and 2014. Despite an overall three-year increase in the age at death, the results showed a significant decrease in hospital costs and use due to reduced interventions and length of hospital stays. However, this was not associated with an increase in quality of life/conditions (as indicated by clinical casemix as a proxy) for end-of-life patients; patients’ casemix characteristics and clinical condition, as measured by the number of comorbidities, disease severity, prevalence of pulmonary disease and heart failure diagnosis, significantly worsened over the decade. This gives rise to important health policy concerns on how to identify effective policies and possible changes in healthcare system organisation to move from hospital-centred care to a community-centred approach whose value has been demonstrated during the COVID-19 pandemic.

## Highlights

• Evolution of costs and stays for patients at end of life.
• Regression and quantile models to assess case-mix heterogeneity.
• Significant decrease in hospital costs and use at end of life.
• No association with increase in quality of life/conditions for end-of-life patients.
• Relevant implications on effective policies and changes in healthcare systems.

## 1 Introduction

In health economics and health policy debate, it is often stated that the ageing of the population will lead to large rises in age-related components of public expenditure (often referred to as the population ageing effect, employment effect, or benefit and eligibility effects; see [1]). Consequently, out-of-pocket expenditure will become important given the increasing risk of poverty at the very end of life of individuals [2]. The need for effective cost-containment policies to ensure sustainability of healthcare systems [3] is particularly relevant considering the demographic transition and population ageing in developed countries – by 2050, 16% of the global population [4] and 32% of the Italian population will be aged over 65 years [5], with a rapidly growing proportion of patients suffering from chronic illnesses.

Based on these considerations, 20 years ago, the World Health Organization (WHO) adopted ‘WHO Health 21′, a health policy recommendation aimed at encouraging health development in 'WHO Member States' for the twenty-first century. This agenda set out global priorities and 21 targets that will create the conditions for people worldwide to reach and maintain the highest attainable level of health throughout their lives [6]. amongst the several targets proposed, two are particularly relevant for the present study. The first target (‘funding health services and allocating resources’) requires that spending on health services must be adequately justified by the real needs of the population, and that funding mechanisms of healthcare systems should guarantee their sustainability. Thus, the importance of monitoring funding allocation and use of resources for health services and care, in coordination with relevant international institutions, has increased amongst developed countries (see [7] for a description of coordinated EU Actions on Sustainability of Health Systems).

From this perspective, previous econometric studies have shown that healthcare utilisation and its related costs are highly concentrated in hospital inpatient services with a corresponding increase at patients’ end of life [8, 9]. First, the peak of individual lifetime healthcare expenditure is concentrated in the last year – or months – of life, independent of the age at which death occurs [8]. Second, patients’ nearness to death is the main driver of hospital costs [10, 11]. Third, the probability of an acute-care hospital admission is high in the last year of life [12, 13, 14]. In the United States, Medicare estimates demonstrate that 25% of healthcare expenditure is attributable to the last year of life, and inpatient care accounts for 40% of medical costs during the last 365 days of life. Other studies confirm that the share of healthcare costs attributable to elderly patients, especially cancer patients, shortly before their death is disproportionate [15, 16], suggesting that end-of-life costs might be reduced by decreasing hospital services in Intensive Care Units (ICUs), especially for terminally ill patients that are particularly resource-intensive [17, 18].

Moreover, reducing the risk of hospitalisation, especially in acute settings, of terminally ill patients has been demonstrated as a relevant issue from a public health perspective. First, the use of the acute hospital system, in particular critical and intensive care services, may have limited potential benefits for patients’ quality of life [19, 20]. In fact, the use of critical and intensive care increases costs but does not always bring the expected benefits, leading to the question of what the real cost-effectiveness is of such services at the end of life [21]. Second, previous studies have found that patients near the end of their life prefer to remain at home [15, 16, 22, 23] while being treated for acute illnesses [24, 25]. Therefore, recent studies have argued that hospital-centred systems should move towards more decentralised systems (community-centred and home-centred) for both non-emergency cases and terminally ill patients, especially the latter. This is important not only for saving public money and avoiding cost increases (balancing resources between health promotion, protection, treatment and care) but also for improving the quality of the healthcare services delivered and the quality of the patient's life [25, 26, 27, 28].

This topic is strongly associated with another ‘WHO Health 21′ target, ‘an integrated health sector’, which is relevant for this paper. In fact, while highlighting a new paradigm for healthcare systems, the WHO recommended moving from a hospital-centred care system to one more community-centred, or more explicitly, towards ‘integrated family- and community-orientated primary health care, supported by a flexible and responsive hospital system’. Specifically, the role of primary healthcare services should be advocated to integrated multidisciplinary teams coordinated by family health physicians and nurses. In this way, the national healthcare system should ensure continuity of care for patients. In addition, an efficient and cost-effective system of referrals to secondary and tertiary hospital services is required for people needing specialist skills and facilities, to ensure that each patient is allowed to die in dignity and to reduce the possible pain, distress and social isolation in hospital settings at the end of life.

This paper used data from public and private hospitals in Lombardy for two cohorts of deceased patients in their last year of life to answer two important research questions: (1) Have end-of-life hospital costs and stay significantly changed over time? (2) How have clinical conditions, the casemix and setting allocation changed over time? The first research question is aimed at measuring for a whole population the hospital costs and utilisation of healthcare services at the very end of life. It will also demonstrate whether or not the healthcare system has been able to increase its efficiency over time, and thereby help to fill a gap in the literature. The objective of the second research question is to provide important evidence on changes in healthcare system utilisation and setting composition at the end of life in order to shed light on whether and how it is possible to move from a hospital-centred care system towards a different paradigm.

To our knowledge, except for some studies reporting essentially descriptive information on per capita costs, both overall and stratified by age class and gender [29, 30, 31], in the last period of life for some Italian regions, very few published studies have described hospital costs and utilisation of healthcare services for a whole population. This partly depends on the lack of information from traditional sources about cost at the end of life and the only available information still being estimated using survey data. Moreover, while a few studies have described use and cost at the very end of life, to our knowledge, none have examined yet the evolution over time of inpatients’ hospital healthcare use and costs in the last year of life by retrospectively following healthcare histories.

This study used data on all hospital discharges in the Lombardy region of Italy to focus on the evolution of healthcare service use and costs in the final year of patients’ life by comparing two different cohorts of deceased patients from 2005 and 2014. The proposed analysis, which focused mainly on cohort effects and identifying the impact of casemix characteristics on use and costs (and casemix composition changes), was conducted with data on the whole population of deceased patients. In this analysis, the data were also disaggregated by healthcare setting (acute care, palliative care and rehabilitation), and was thus able to retrospectively shed light on the overall impact of ongoing institutional changes in the healthcare system in the decade of interest.

## 2 Materials and methods

We conducted a retrospective cohort study to analyse healthcare use and costs arising from hospitalisation of inpatients in their last year of life in Lombardy region (Italy). The main characteristics of the Lombardy healthcare system are briefly described in the Appendix.

We analysed all patients who died in two distinct periods: the first covering hospitalisations from 1 January to 31 December 2014; and the second from 1 January to 31 December 2005. In each period, we tracked the patients’ paths in the 365 days prior to death and observed both hospitalisations (in public and private hospitals) and their related costs.

We chose these two cohorts for several reasons. We wanted to consider the 10-year gap between the cohorts in order to observe differences in expenditure (in terms of composition and casemix) over time, given the evolution of population needs across an entire decade. Furthermore, choosing 2014 to end the decade of study allowed us to avoid a recent major change in the rules adopted by the Lombardy healthcare system in 2015 (pointing towards a more collaborative system with the creation of hospital networks for the treatment of acute pathologies and incentivising associations amongst general practitioners for the management of chronic patients); the rule change would have made comparison of hospital reimbursements with the previous period inappropriate.

Healthcare use and costs were computed according to hospital discharge reimbursement charts which captured both clinical and demographic information for inpatient care (hospital admissions, diagnosis-related groups [DRGs], ICU admissions, emergency department visits, up to six diagnoses, and intervention codes). Each hospitalisation record collected both the length of hospital stays and the amount reimbursed to the hospital by the healthcare purchaser (Lombardy region) according to specific tariffs defined each year by the regional prospective payment system based on the DRG classification of discharges. To ensure comparability, all 2005 costs were adjusted to 2014 prices using the healthcare-specific yearly Consumer Price Index reported by the Italian Institute of Statistics - ISTAT. Inpatient hospital stays and healthcare expenditure for the whole population were then calculated as the sum of all hospital stays and costs generated for deceased patients in each setting, the units of analysis.

Moreover, for reliable comparison of the 2005 and 2014 cohorts, we excluded patients affected by cancer since a change in oncological reimbursement rules occurred in the analysed period: in 2005, oncological patients were regarded as acute patients (e.g. they were often admitted to day hospital), whereas from 2008 onwards, they were categorised as outpatients, which made the two cohorts not fully comparable. In particular, we filtered out deceased patients with cancer as their main cause of death according to the International Classification of Diseases, Ninth Revision (ICD9). The following diagnoses were then excluded from the analysis: breast [ICD9–174], lung [ICD9–162], colorectal [ICD9–153 and ICD9–154], prostate [ICD9–185], haematologic [ICD9–196 and CD9–200 to ICD9–208], and other [all other ICD9 cancer codes]. Similarly, in order to ensure a fair comparison of healthcare practices in place between 2005 and 2014, we excluded all daily hospitalisations from our analysis. Finally, we also excluded patients hospitalised in Lombardy but living in other Italian regions (15% of the total number of discharges) because their healthcare costs during the last year of life could not be fully accounted for without considering the costs incurred in their region of origin.

To study the differences in both hospital stays and costs for the healthcare system between 2005 and 2014, we considered (log of) reimbursement/cost and (log of) length of hospital stays as our primary outcomes of interest. Healthcare costs were measured as the annual reimbursement for patients’ hospitalisations based on the DRG prospective payment system, whereas the length of hospital stays was computed considering the number of days between the date of admission and the date of discharge of each episode of hospitalisation. The two outcomes were modelled, after controlling for available patient covariates to account for heterogeneity amongst patients, using a linear regression with ordinary least squares (OLS) estimator and standard errors clustered at the patient level, in order to take into account the annual costs of different types of hospitalisation which were nested within patients.

Available covariates at patient level included individual demographic characteristics (*sex, age*), the number of hospitalisations during the year, the number of comorbidities (identified using the Elixhauser algorithm [32]), a proxy for disease severity provided by the DRG weight (*DRG weight*), the type of hospitalisation (*surgical/medical*), admission type (*emergency/planned*), and dummy variables for the following chronic conditions: chronic obstructive pulmonary disease (COPD, ICD9-CM = 490–496), diabetes (ICD9-CM = 250xx), heart failure (ICD9-CM = 428xx) and others conditions.

We considered the hospitalisation setting as the stratification variable as patients’ hospitalisations were categorised by the hospital discharge chart classification as acute, rehabilitation and palliative care. Specifically, palliative care referred to patients admitted to palliative care units (code ‘99′), corresponding to four DRG codes (MS-DRG 24th version, 2007): DRG 467 (‘other factors influencing health status’); DRG 463/464 (‘signs and symptoms with or without complications’); and DRG 414 (‘other myeloproliferative disorders or poorly differentiated neoplastic diagnosis without complications’). Rehabilitation settings corresponded to three specialties: intensive rehabilitation (code ‘56′); extensive rehabilitation (code ‘60′); and high-intensive rehabilitation (code ‘75′). Such hospitalisations, typically referring to patients being transferred from acute specialties, corresponded to four major diagnostic categories: diseases and disorders of the nervous system; diseases and disorders of the respiratory system; diseases and disorders of the circulatory system; and diseases and disorders of the musculoskeletal system and connective tissue. Finally, acute setting corresponded to the remaining hospitalisations.

The main coefficient of interest of the estimated regression models was the dummy variable related to the year/cohort analysed (2014 vs 2005), which provided an estimate of the difference between the two cohorts in the annual length of hospital stays and overall costs, after controlling for patients’ covariates. The regression analyses of both log-transformed dependant variables for patients were performed for the whole set of annual hospitalisations regardless of their hospital setting (‘pooled model’) and after stratifying by hospital setting (acute, rehabilitation or palliative). Moreover, the same four models were fitted separately only for the last cohort (‘2014 model’) in order to evaluate whether (i) casemix characteristics (covariates) had a significant impact in the most recent cohort; and (ii) covariate effects were different from the effects obtained with the pooled model.

However, regression models estimate the effects of the covariates at the mean of the dependant variable and, given the properties of OLS estimators, they may be affected by deviations from the underlying assumptions (e.g. linearity and absence of skewness and outliers). As a robustness check for OLS, we re-estimated our parameters by adopting a quantile regression estimator [33] which modelled the relationship between covariates and the dependant variable at different percentiles of its conditional distribution (quantiles). A quantile regression was specified to study the cohort effect on the entire quantile regression process (the pattern of coefficients estimated for all quantiles of the dependant variables considered) in the pooled model, using the same covariates included in the OLS models. Confidence intervals (95%) for the coefficients of regression quantiles, obtained using bootstrap resampling (200 replications), were used to assess the significance of the estimated cohort effects and areas (quantiles) of statistical significance.

## 3 Results

Table 1 presents descriptive statistics for the overall costs, length of stay and casemix in the last year of life for the population as a whole and by hospital setting. We observed 78,926 deaths in 2014^1^ and 67,172 in 2005, with at least one healthcare record for the deceased patients in their last year of life. Overall average cost of inpatients in the last year of life was €9916 in 2014 (leading to a total cost of €1.191 billion), a decrease from €10,120 (the total cost was €1.265 billion) in 2005. Acute costs contributed about 86% to the total costs, and even when acute hospitalisations accounted for a similar share of the total costs in the two cohorts, the percentage of acute users increased over time (78% in 2014 versus 69% in 2005), significantly reducing the mean cost per user (the mean difference was close to €1600, *p* < 0.0001). In addition, the average cost of inpatient rehabilitation of users was higher (only acute care was more costly) and significantly increased over time (+€649, *p* < 0.001); the proportion of users also increased (+3.2%). Furthermore, only a small percentage of patients in the two cohorts (5% in 2005 and 8% in 2014) received palliative care at a similar (not significantly different) average annual cost (approximately € 4900 in 2014 and € 5200 in 2005).

**Table 1:** Comparison of costs, length of hospital stays (LoS) and cohort casemix over time, by setting.

		2014	2005	Difference
Variable	Group	Mean	Mean	2014–2005
Cost	Total	€ 9916	€ 10,120	-€ 204
	Acute	€ 10,898	€ 12,479	-€ 1580***
	Pall. Care	€ 4856	€ 5230	-€ 373
	Rehab.	€ 10,055	€ 9406	€ 649***
LoS	Total	14.3	17.4	−3.1 ***
	Acute	12.6	12.2	0.4 *
	Pall. Care	16.6	19.7	−3.1 ***
	Rehab.	27.8	24.2	3.6 ***
%Male	Total	52.6%	54.4%	−1.9%***
	Acute	51.7%	52.9%	−1.2%***
	Pall. Care	54.7%	54.9%	−0.30%
	Rehab.	53.8%	54.3%	−0.50%
Age	Total	78.1	74.4	3.6***
	Acute	78.7	75.9	2.8***
	Pall. Care	74.1	71.9	2.2***
	Rehab.	79.2	76.6	2.6***
N. of Comorbidities	Total	0.6	0.2	0.4***
	Acute	0.6	0.2	0.4***
	Pall. Care	0.8	0.3	0.5***
	Rehab.	0.9	0.3	0.6***
DRG Weight	Total	1.5	1.1	0.3***
	Acute	1.6	1.2	0.5***
	Pall. Care	0.6	0.8	−0.2***
	Rehab.	1.0	1.0	0
%Emergency	Total	13.4%	12.3%	1.2%***
	Acute	16.9%	17.2%	−0.30%
	Pall. Care	0.0%	0.0%	0.0%***
	Rehab.	0.0%	0.0%	0.0%***
%Surgery	Total	15.8%	11.2%	4.5%***
	Acute	18.4%	12.4%	5.9%***
	Pall. Care	0.0%	0.6%	−0.6%***
	Rehab.	0.1%	0.2%	−0.10%
%COPD	Total	1.6%	1.4%	0.2%***
	Acute	1.6%	1.5%	0.1%***
	Pall. Care	0.2%	0.4%	−0.20%
	Rehab.	2.6%	2.4%	0.20%
%Heart failure	Total	7.8%	7.0%	0.8%***
	Acute	8.6%	8.0%	0.5%***
	Pall. Care	0.6%	2.5%	−1.9%***
	Rehab.	9.3%	8.3%	0.9%***
%Diabetes	Total	0.5%	0.6%	−0.10%
	Acute	0.6%	0.6%	−0.10%
	Pall. Care	0.1%	0.2%	−0.2%**
	Rehab.	0.4%	0.3%	0.10%
N. of Hospitalizations	Total	1.99	2.17	−0.18
	Acute	2.2	2.3	−0.1***
	Pall. Care	1.07	1.14	−0.06***
	Rehab.	1.27	1.28	−0.01

*Note:* *** *p* < 0.001, ** *p* < 0.005, * *p* < 0.01.

The mean length of hospital stays in 2014 was 14.3 days (12.6 days for acute inpatients and 27.8 days for rehabilitation inpatients), which was significantly shorter than the 17.4 days in 2005 (*p* < 0.001). Acute patents in 2014 had the largest share in total length of stay (69%), which increased strongly over time (from 48% in 2005). In the case of patient characteristics in the final year of life (see Table 1), males and females were almost equally distributed in the two cohorts.

The deceased patients were older in 2014 (an average age of 78.1 years compared to 74.4 years in 2005), and other casemix characteristics - average number of comorbidities, mean disease severity (DRG weight), percentage of surgical interventions, and COPD and heart failure diagnosis as expected for an ageing population - showed a worse clinical outcome for the 2014 cohort.

The average number of hospitalisations for inpatients was 2.0 days in 2014 (not significantly different from that in 2005 at 2.2 days) and ranged from 1.1 days (palliative care inpatients) to 2.2 days (acute inpatients). Acute patients (not shown in Table 1) accounted for the majority of hospitalisations in 2014 (86%), with a strong increase relative to 2005 (73%), whereas the proportion of palliative care hospitalisations increased over time from 2.8% to 4.8% of all hospitalisations.

Table 2 shows the main results for regression models of log-transformed reimbursements. Estimated parameters are reported as the adjusted mean differences between the two cohorts, both for all settings (column 2 ‘pooled’) and within each setting (acute, palliative care and rehabilitation in columns 4, 6, and 8, respectively).

**Table 2:** Regression results for the dependant variable, the log of reimbursements (OLS with clustered standard errors).

	All settings		Acute		Pall. Care		Rehabilitation	
	Pooled model	2014 model	Pooled model	2014 model	Pooled model	2014 model	Pooled model	2014 model
Observations	174,015	96,376	144,237	77,786	12,546	8131	17,232	10,459
2014 (vs 2005)	−0.189***		−0.219***		−0.150***		0.034**	
	0.004		0.004		0.022		0.016	
Male	0.022***	−0.006	0.051***	0.023***	−0.230***	−0.257***	−0.017	0.001
	0.004	0.005	0.004	0.004	0.020	0.025	0.014	0.018
Age	−0.004***	−0.002***	−0.004***	−0.001***	−0.001	−0.002	−0.002**	−0.003***
	0.000	0.000	0.000	0.000	0.001	0.001	0.001	0.001
N. of Hospitalizations	0.369***	0.399***	0.361***	0.391***	0.976***	1.075***	0.597***	0.609***
	0.002	0.002	0.002	0.002	0.024	0.034	0.011	0.013
N. of Comorbidities	0.080***	0.089***	0.091***	0.107***	0.039***	0.024	0.029***	0.038***
	0.003	0.003	0.003	0.003	0.015	0.018	0.009	0.009
DRG Weight	0.159***	0.181***	0.159***	0.180***	−0.019	−0.089	0.182***	0.172***
	0.002	0.002	0.002	0.002	0.045	0.079	0.023	0.026
Emergency	0.147***	0.116***	0.161***	0.124***	−0.316***	−0.486***	−0.298***	−0.170**
	0.007	0.007	0.007	0.007	0.113	0.174	0.053	0.085
Surgery								
(vs Medical)	0.725***	0.787***	0.735***	0.797***	0.572***	1.027*	−0.127	−0.152
	0.007	0.008	0.007	0.008	0.184	0.559	0.180	0.240
Heart failure (vs COPD)	−0.008	−0.005	−0.011	0.002	−0.123	−0.277	0.008	−0.081
	0.014	0.016	0.013	0.014	0.224	0.360	0.047	0.057
Diabetes (vs COPD)	−0.369***	−0.336***	−0.420***	−0.379***	0.221	0.586	0.062	−0.100
	0.027	0.032	0.026	0.029	0.272	0.378	0.140	0.170
Others (vs COPD)	−0.043***	−0.087***	−0.071***	−0.117***	0.123	0.212	0.106**	0.058
	0.013	0.015	0.012	0.013	0.209	0.320	0.044	0.053
Acute (vs Palliative)	0.284***	0.185***						
	0.011	0.014						
Rehab. (vs Pall. Care)	0.745***	0.786***						
	0.012	0.016						
Constant	7.830***	7.466***	8.174***	7.644***	6.984***	6.786***	7.884***	8.053***
	0.023	0.027	0.022	0.025	0.224	0.337	0.083	0.101
R-squared	0.440	0.535	0.484	0.623	0.114	0.102	0.156	0.151

*Note:* *** *p* < 0.001, ** *p* < 0.005, * *p* < 0.01.

The results reported in columns labelled ‘2014′ refer to a model estimated only with 2014 data to assess covariate and casemix effects on log-costs when the most recent cohort/period was considered (for all settings and by each setting separately). The results of the pooled model showed that, assuming €11,000 as the reference level for the average cost of the two cohorts, the average patient cost in the last year of life was significantly reduced by 17.2% (= *e* ^− 0.189^− 1) over the decade. This result seems to be driven by a significant reduction in the expenditure on acute (−19.7%) and palliative care (−13.9%); this is in contrast to expenditure on rehabilitation (which also included long-term care) which increased significantly (+3.5%). Casemix variables showed that one additional comorbidity increased the annual patient cost by 8.3%, while an additional hospitalisation had an impact of +44.5% (surgical intervention costs were twice the medical hospitalisation costs [+107%]) on annual patient costs. COPD and heart failure (which were not significantly different) were the most expensive diagnoses (diabetes costs were 31% less than the COPD costs), while acute care was the most expensive setting for patients at end of life (33% more than palliative care). Such overall patterns were confirmed for the settings, especially for acute, except for the following: for palliative care, for which no significant differences between diagnoses emerged, one additional hospitalisation increased the cost by 165%, while a surgical intervention cost 77% more, but less than the overall pattern (107%).

Table 3 provides the same comparison for log-length of hospital stays. Considering an average stay of 16 days as the reference value for the two cohorts, the mean length of stay significantly reduced over time (−3.1%, like acute setting), but showed high variability amongst settings (−22% for palliative care and +14% for rehabilitation). The length of stay of patients from the two cohorts in acute settings was 18% shorter compared to that of patients being treated in palliative care settings, while for rehabilitation settings, the length of stay was 75% longer than that in palliative care settings. COPD patients (differing little from patients with heart failure) stayed longer than other diagnostic groups (7.7% more than diabetes) overall, but this pattern was reversed for the palliative care setting (−14.1%). Hospital stays in palliative care settings were longer (+38.8%) than the overall pattern (+2.9% and +3.4%, respectively) especially for surgical interventions (+53.1%).

**Table 3:** Regression results for the dependant variable, log of length of stay (OLS with clustered standard errors).

	All settings		Acute		Pall. Care		Rehabilitation	
Covariates	Pooled model	2014 model	Pooled model	2014 model	Pooled model	2014 model	Pooled model	2014 model
Observations	173,499	96,226	143,858	77,681	12,530	8127	17,111	10,418
2014 (vs 2005)	−0.031***		−0.031***		−0.247***		0.132***	
	0.004		0.004		0.019		0.013	
Male	−0.014***	−0.025***	0.005	−0.002	−0.206***	−0.225***	−0.028**	−0.027*
	0.004	0.005	0.004	0.005	0.017	0.021	0.011	0.015
Age	−0.000**	0.001**	−0.000**	0.001***	0.001	−0.001	−0.002***	−0.001
	0.000	0.000	0.000	0.000	0.001	0.001	0.001	0.001
N. of Hospitalizations	0.029***	0.042***	0.026***	0.040***	0.328***	0.404***	0.047***	0.038***
	0.001	0.001	0.001	0.002	0.018	0.026	0.007	0.009
N. of Comorbidities	0.108***	0.120***	0.130***	0.148***	0.036***	0.022	0.034***	0.039***
	0.003	0.003	0.003	0.004	0.013	0.016	0.007	0.008
DRG Weight	0.077***	0.082***	0.075***	0.082***	0.116***	−0.063	0.024	0.021
	0.002	0.002	0.002	0.002	0.039	0.067	0.018	0.020
Emergency	−0.178***	−0.210***	−0.177***	−0.211***	−0.211**	−0.445***	−0.157***	−0.069
	0.006	0.008	0.006	0.008	0.100	0.138	0.040	0.064
Surgery	0.033***	0.033***	0.037***	0.042***	0.426**	0.895*	0.474***	0.599***
	0.008	0.010	0.008	0.010	0.178	0.526	0.165	0.149
Heart failure (vs COPD)	−0.027**	−0.021	−0.024*	−0.016	−0.107	−0.278	−0.051	−0.076*
	0.013	0.016	0.013	0.017	0.197	0.303	0.037	0.046
Diabetes (vs COPD)	−0.080***	−0.084***	−0.105***	−0.113***	0.128	0.431	0.110	0.003
	0.024	0.031	0.024	0.032	0.238	0.328	0.103	0.122
Others (vs COPD)	−0.026**	−0.037**	−0.036***	−0.059***	0.020	0.143	0.047	0.060
	0.012	0.015	0.012	0.015	0.184	0.271	0.033	0.042
Acute (vs Pall. Care)	−0.197***	−0.115***						
	0.009	0.012						
Rehab. (vs Pall. Care)	0.557***	0.688***						
	0.010	0.013						
Constant	2.428***	2.223***	2.227***	2.068***	2.243***	2.074***	2.998***	3.074***
	0.020	0.026	0.020	0.027	0.197	0.285	0.064	0.079
								
R-squared	0.122	0.156	0.053	0.089	0.046	0.035	0.017	0.008

*Note:* *** *p* < 0.001, ** *p* < 0.005, * *p* < 0.01.

## 4 Variability in cohort effects

Quantile regression (QR) can help to understand the observed reduction in hospital costs and stay between 2005 and 2014 in the last year of life (cohort effect) and whether cohort effects could be more evident for high users (most expensive or longest staying patients, e.g. patients in ICUs or with longer lengths of stay in hospital or with a relevant number of hospital admissions during the year) or for medium- or low-user categories.

**Fig. 1:**
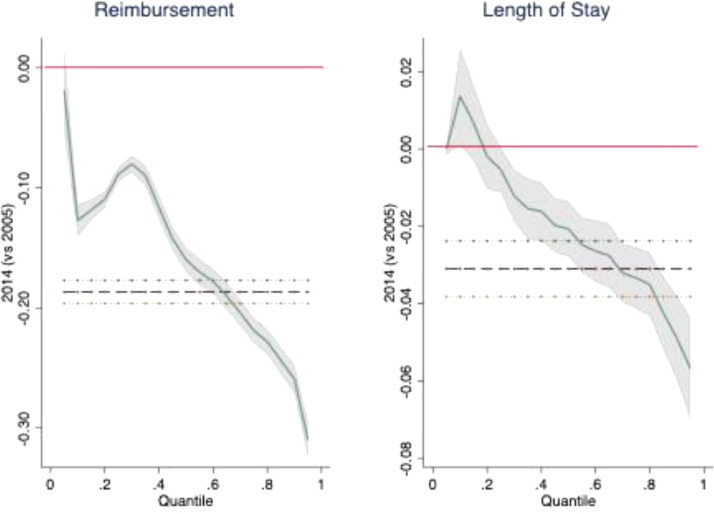
Quantile regression of the log of dependant variables showing the cohort effect (2014 vs 2005).

Fig. 1 reports coefficient estimates for cohort effects (with the shaded area indicating the 95% confidence interval) conditional on covariates at different deciles of each dependant variable. Horizontal red lines identify the null effects, whereas dashed black horizontal lines represent the OLS coefficients and 95% confidence intervals, to be compared with the coefficients in the quantile regression. It shows that the higher the decile of health consumption (cost and stay) the larger the reduction in costs and use in 2014 compared to 2005: this effect (at each decile) was always statistically significant for costs, as well as for hospital stays, except in the first three deciles (where the parameter confidence intervals crossed the y-axis at zero). This supports the idea that, over time, the Lombardy healthcare system was able to increase its efficiency and the appropriateness of the services provided by making larger reductions in the overall cost and length of hospital stays of the average ‘patient’, and of the costlier and longer-hospitalised patients in particular, rather than of the less expensive and short-stay patients.

## 5 Discussion

Our results showed a decrease in the average patient cost and length of hospital stays during the last year of life in 2014 compared to 2005. These effects were statistically significant also after adjustment for potential confounding factors as well as age, sex, comorbidities, disease severity, main chronic condition and setting. In particular, the average cost of inpatients in the last year of life was € 9916 in 2014 (a decrease from € 10,120 in 2005), and ranged from € 4856 for inpatient palliative care to € 10,898 for more expensive acute patients. Our study extends the evidence from the international literature, mainly for the Canadian context and documenting the use and costs of healthcare services in the last year of life in different hybrid settings (by mixing different healthcare services/settings). Compared to a 2004–2006 British Columbia study [34] and a 2003–2004 Saskatchewan study [35], which provided estimated average expenditure in the last year of life ranging from $20,705 to $31,492 Canadian dollars (€13,816 to € 21,020), we found a lower cost per deceased patient. However, a clear comparison with these estimates is not straightforward considering that the Canadian studies used different patient inclusion criteria or healthcare services; in fact, the first study examined hospital, ambulatory, and prescriptions drug costs, while the second included long-term care and home care. In addition, a 2010–2013 Ontario study [36] estimated the last-year-of-life costs of all deceased patients for all healthcare services and found an average cost of inpatient care of $30,872 (€20,573). The difference between our results and the findings of previous studies can be partially explained by the exclusion of cancer patients at end of life in our study. In this context, other previous studies have analysed end-of-life healthcare costs by focusing on specific target patients in the US population, for example, adults aged ≥ 65 years or selected disease-specific cohorts, such as cancer patients and patients with heart failure [16, 37, 38, 39, 40, 41]. Again, the different populations studied make a clear comparison with our estimates difficult.

However, the reduction in costs and hospital stays do not entirely explain the broad picture. The contribution of acute patient hospitalisations (+13%), which accounted for 86% of the costs in the most recent cohort, greatly increased over time. Moreover, the number of hospitalisations showed a significant impact on costs and length of stay, and such effects increased over time for all settings, especially for palliative care, after adjustment for potential confounding factors.

It is worth noting that any decrease in hospital activity, as a result of reduced hospital interventions, the length of hospital stays and number of hospitalisations, should correspond to an improvement in decentralised healthcare provision at both regional level (in terms of continuity, integrated primary care services, community care and social care) and patient level (accessibility and quality of life) in the last year of life. However, our results are incompatible with this proposition. Although we observed a reduction in hospital stays and costs, the Lombardy healthcare system seems to have progressively focused more on acute patients usually showing worse clinical conditions than patients in other settings (especially in terms of prevalence of surgical interventions and COPD and heart failure treatments). In fact, the reduction in hospital activities over time was associated with worsening casemix conditions at patient level, which can be partially explained by the increase in patient age. Moreover, over time, we found an increase in the percentage of acute users and hospital admissions (out of all users) as well as the contribution of acute care to the total length of stay. In addition, only a small percentage of patients received palliative care in their last year of life (even when the share of palliative care hospitalisations in all hospitalisations increased over time), while many studies have demonstrated increasing proportions of adults and non-cancer and paediatric patients needing palliative care in the European population [42, 43, 44]. The WHO has estimated that in Europe, about 562 adults/100,000 could benefit from palliative care, meaning that about 300,000 Italian patients (0.56% of the total) every year should have access to palliative care, while recent estimates [45] suggest that no more than 80,000 patients (0.15% of the total) receive it.

In this study, although the mean length of hospitalisations in palliative settings significantly reduced over time, palliative care still showed longer hospital stays (an effect that is more evident in the 2014 cohort), especially for surgical-type hospitalisations and more often for patients with diabetes and other diagnosis, than the overall pattern, demonstrating that emergencies of terminally ill patients were essentially managed with urgent hospital admissions and longer stays. The same pattern was confirmed for acute patients, the deterioration of whose risk factors (typically the number of comorbidities, DRG severity and COPD diagnosis), which required hospitalisation, was significantly associated with longer hospital stays. This evidence is compatible with the still scarce home/community care programmes in the region. Community care programmes that are integrated with primary care and hospitals would be better able to manage terminally ill patients within a comprehensive approach (through optimal symptom management and planned specialised multidisciplinary interventions) which may have the potential to improve patient-centred outcomes and increase their quality of life [25, 26, 27, 28].

A possible cause for such intensive use of hospitalisation may be the adoption in many hospitals of life-preserving therapies, which often consist of aggressive care interventions. However, empirical evidence demonstrates that a higher intensity of care at the end of life does not necessarily lead to better outcomes [46, 47]. Cutler and colleagues [48] reported that the intensity of care mainly depends on ‘physician beliefs about the efficacy of certain therapies’ and that these beliefs are often not consistent with professional guidelines for appropriate care or justified by clinical effectiveness. Different views amongst physicians indicate a diversity of opinions on how to treat patients and explain the variability in more than a third of end-of-life Medicare expenditures, whereas patient preferences have only a small impact on expenditure variability.

Although there is no formal consensus on the best practices for adoption at the end of life [49], recent interdisciplinary studies provide some evidence. Aggressive care is considered to be suboptimal at the end of life; instead, alternative care settings, such as home (palliative) care, may improve quality of life [50, 27, 28], while at the same time avoiding hospital admissions, leading to associated cost reductions [51, 52, 53]. From an economic point of view, savings on institutional and outpatient services tend to exceed the cost of increasing home and community care services [54]. In a systematic review of different care settings in the last year of life, palliative care was frequently found to be the less costly alternative to other settings [55] and an efficient way of allocating health resources and expenditure [56, 57].

Notwithstanding the increasing evidence from the literature, end-of-life care is ignored in discussions of healthcare reform, resulting in the vast majority of patients at the end of life not receiving high-quality care at their homes, hospitals, or long-term care facilities [58, 59, 60]. A noticeable exception from Italy is the statement of the first Consensus Conference on the care for patients with chronic complex, advanced conditions and limited life expectancy that all people with advanced and progressive chronic conditions may benefit from a community-integrated palliative care approach. From a managerial point of view, this approach involves early patient identification which is associated with better needs assessment and flexible diagnostic plans shared by an integrated group of professionals (family health physicians and/or multidisciplinary teams). This approach is a way of effectively implementing one of the main targets (‘an integrated health sector’) of the Health-21 agenda.

These issues seem to have become even more central during the current COVID-19 pandemic, with hospitals in many parts of the world required to operate at crisis capacity despite a large proportion of patients (> 80%) developing only mild to moderate symptoms without the need for hospitalisation [61, 62]. Instead, during the pandemic, Italian hospitals suffered dramatic reductions in their daily activity, such as urgent surgery and oncological programmes [63, 64], acute myocardial infarction treatments [65], and, in a holistic context, mental health therapies [66] and stroke interventions [67].

The transition towards community-centred care, as proposed by the Alma-Ata definition [68] aimed at the creation of multidisciplinary care facilities inspired by the core principles of primary healthcare [69], has not yet been reached, as dramatically evidenced during the COVID-19 pandemic [70, 71, 72]. For healthcare systems, it is crucial to understand during ‘normal’ times how best to balance costs and savings – by increasing community and home care services accompanied by a reduction in hospital-based services when appropriate – to deal with potentially extraordinary expenditure during epidemic outbreaks.

### 5.1 Strengths and limitations

A strength of this paper is the new information it provides about the breadth of healthcare costs in the last year of life for two cohorts of hospitalised citizens in the Lombardy region. Moreover, we showed how registry data can be useful for effectively monitoring the use of services and healthcare expenditures in the last year of life of patient subgroups (by settings and low–high users), including from a cross-cohort perspective. This approach was able to shed light on how the Lombardy healthcare system was able to increase its efficiency and the appropriateness of the care provided.

However, this paper also has several limitations. First, the analysis covered only one region. Second, we excluded cancer patients. These aspects limited the analysis, preventing a detailed picture of all patient groups. In particular, we excluded cancer patients cared for outside hospital settings, which restricted the study to an analysis of the evolution of use and costs only for other diseases. Third, we did not consider the use and costs of outpatient settings outside hospitals, such as community-based settings, psychiatric hospitals, residential facilities and home-care settings. Such services together are estimated to contribute to about 20% of the total costs (see the Ontario study [36]).

From this perspective, a comprehensive evaluation of costs and expenditure should also include informal (caregiver) care [55], such as time spent assisting patients with daily activities, medications and administrative tasks, which accounts for a high proportion of costs during patients’ last year of life [73]. For example, a recent study [74] quantified formal and informal costs in 2010 for a sample of patients who received specialist palliative care in three different areas of Ireland; informal costs amounted to more than 22% of the total expenditure in the last year of life, and this rate did not vary across geographical areas and organisational models. New approaches should be pursued to analyse in detail the benefits and possible trade-offs (cost–benefits) of care at the end of life.

## 6 Conclusion

This paper demonstrates an effective use of existing archives to shed light on under-investigated research questions, such as how end-of-life healthcare use and costs may have changed within a decade. Indeed, the results of this study, as long as the last evidence from the ongoing COVID-19 pandemic shows, confirm that it is essential to study the evolution of individual patterns of healthcare use and costs. Such studies may suggest effective ways to reduce healthcare expenditure and to focus on more appropriate care/settings to ensure a better quality of life for patients in the last days of their life. Therefore, increasing social care and improving integration between hospitals, outpatient settings and home care services will pose new challenges to the safe and timely reaction to unforeseen disease outbreaks and preparation of healthcare systems to deal with these unexpected additional stresses.

Retrospectively, the strong impact of the COVID-19 pandemic on the Lombardy region, in terms of additional stress on hospitals and ICUs, an exponential increase in infective cases and hospitalisations, infection in hospital care workers and nursing homes, has moved public opinion, health managers and relevant experts to call for a new paradigm for the regional healthcare system, from one that is more hospital-centred to a new community-centred approach [75]. To conclude, although it is difficult to identify what proportion of hospital costs could be reduced by the adoption of more appropriate treatments or by the introduction of interventions that reduce or delay institutional care, more studies are needed to monitor factors, such as physician treatment beliefs and patient preferences, that may drive temporal and geographical variation in treatment and healthcare spending at the end of life.

### Contributors

All authors contributed equally to the statistical analysis and the writing of the manuscript.

### Role of funding source

No funding source received for this study.

### Ethics committee approval

Not required.

## Declaration of Competing Interest

We declare no competing interests.

## Appendix

The Italian national healthcare system (NHS) provides universal healthcare coverage funded mainly from general taxation. The state government guarantees essential levels of assistance (LEA) to all regions of the country. Financial resources for the NHS are transferred from the state to regional budgets and are then managed by the local healthcare system. amongst the 21 regions in Italy, Lombardy is one of the top-ranked for socio-demographic indicators and one of the most competitive areas in Europe according to economic indicators. Lombardy has a population of 10 million, equal to 16% of the total Italian population, with a density of 404 inhabitants per km^2^. The Lombardy healthcare system includes approximately 150 hospitals with about 1.7 million discharges per year, and spends 18 billion euros (circa 75% of the regional budget). Regional reform in 1997 radically transformed the healthcare system in Lombardy to a quasi-market system in which private for-profit, private not-for-profit and public hospitals compete with each other, and citizens can freely choose providers for hospitalisation. The 1997 reform also established that the Lombardy administration is responsible for monitoring the effectiveness of the healthcare provided by the hospitals belonging to the regional accreditation system [76]. The healthcare system in Lombardy is entirely built on a prospective payment system based on DRGs, with a maximum annual reimbursement defined by a budget allocated annually by the region to each hospital [77]. Each DRG is reimbursed according to a tariff defined at the beginning of each year. Consequently, DRG tariffs can become health policy leverage that may favour or disincentivise specific DRGs. In addition, DRG tariffs may also allow the regional government to restrain healthcare expenditure.

In the case of costs and chronic patients in 2014, the regional government spent about 10.4 billion euros for inpatients (48%), outpatients (25%) and pharmaceuticals (27%). Most of this expenditure was for chronic conditions (which accounts for about 70% of the total expenditure for citizens aged under 65; it increases to 82% for citizens aged over 65), with little difference in the spending between men and women [29]. Although deceased patients were only 0.93% of the Lombardy population in 2014, they incurred nearly 7.3% (around 753 million euros) of the total healthcare costs. More than 79% of the total expenditure for deceased patients (753 million euros) was attributable to inpatient costs (nearly 592 million euros), and per capita inpatient expenditure for dead patients was more than 10 times those for surviving patients in 2012.

## Footnotes

1 The Italian National Statistical Institute reported 92,894 deaths in Lombardy in 2014, which means that 85% of the deceased citizens had at least one hospitalisation in 2014.
